# Mechanisms, advances, and challenges of immunotherapy in gastric cancer

**DOI:** 10.3389/fimmu.2025.1639487

**Published:** 2025-09-04

**Authors:** Zixin Wang, Tengjiao Wang

**Affiliations:** ^1^ College of Basic Medical Sciences, Naval Medical University, Shanghai, China; ^2^ Research Center of Translational Medicine, Naval Medical University, Shanghai, China

**Keywords:** gastric cancer, immunotherapy, immune checkpoint inhibitors, adoptive cell therapy, biomarkers

## Abstract

Gastric cancer (GC) is one of the most common gastrointestinal malignancies worldwide, characterized by a high incidence rate, low screening rate, and subtle early symptoms. As a result, the majority of patients are diagnosed at an advanced or metastatic stage, contributing to poor overall prognosis. In recent years, the emergence and continuous advancement of immunotherapy have revolutionized the traditional treatment landscape for GC, offering new hope for precision medicine. Immunotherapy exerts its antitumor effects primarily by modulating the immunosuppressive tumor microenvironment and includes modalities such as immune checkpoint inhibitors (ICIs), adoptive cell therapies (ACTs), and cancer vaccines. Among these, ICIs and ACT have garnered significant attention. This review summarizes the underlying mechanisms, current applications, and major challenges of immunotherapy in GC. In addition, we discuss emerging biomarkers with potential utility for predicting immunotherapeutic efficacy in GC patients.

## 1 Introduction

Gastric cancer (GC) ranks as the fifth most common malignancy and the fourth leading cause of cancer-related death worldwide (1). Early-stage GC is often asymptomatic, and by the time symptoms manifest, the disease has usually progressed to an advanced stage, resulting in a generally poor prognosis. At this point, surgical resection often fails to achieve a curative outcome, and conventional systemic therapies remain the primary option for prolonging survival (2).

In recent years, remarkable progress in research and clinical trials of immunotherapy has begun to reshape the treatment landscape for GC, offering renewed hope for personalized and precision-based interventions. Building on these advances, this review will focus on the mechanisms, clinical applications, and challenges associated with three major immunotherapeutic strategies in GC: immune checkpoint inhibitors (ICIs), adoptive cell therapies (ACTs), and cancer vaccines. In addition, the use of biomarkers to predict the efficacy of immunotherapy has emerged as a critical area of investigation. Herein, we review the underlying mechanisms, recent advances, and ongoing challenges in GC immunotherapy, with particular emphasis on the development of ACTs and predictive biomarkers.

## 2 ICIs

**Figure 1:**
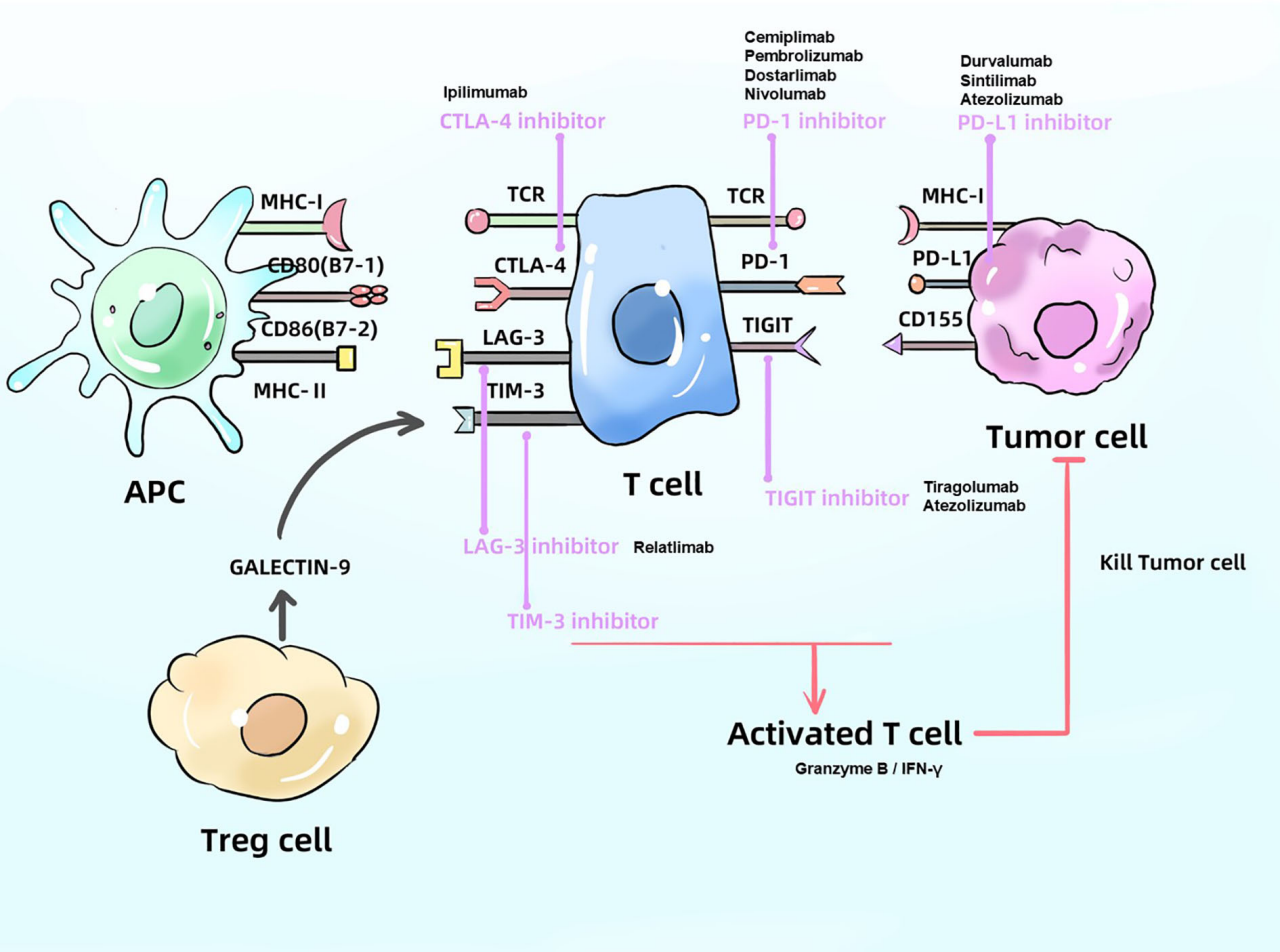
Mechanism of ICIs. ICIs block the proteins like PD - 1、CTLA4 and LAG - 3, produced by cancer cells and APC in order to activate T cells, which can kill tumor cell. APC, antigen presenting cell; PD - 1, programmed death-receptor 1; PD-L1, programmed death-ligand 1; CTLA4, cytotoxic T-lymphocyte associated protein 4; MHC, major histocompatibility complex; TCR, T cell receptor.

Immune checkpoints, often described as “immune brakes,” are inhibitory regulators within the immune system that maintain self-tolerance and prevent damage to host tissues by suppressing excessive immune responses. Within the tumor immune microenvironment (TIME), tumor cells can aberrantly express ligands that engage immune checkpoint receptors (such as PD - 1, CTLA - 4, and LAG - 3) on T cells, thereby restricting immune activity and promoting immune evasion. ICIs function by blocking these inhibitory signals, effectively releasing the “immune brakes” and restoring T cell recognition and cytotoxicity against tumor cells. This section focuses on the mechanisms and recent advancements in various ICIs (**Figure 1**).

### 2.1 Programmed death-1 (PD - 1)/PD-L1

In recent years, the PD - 1/PD-L1 pathway remains a central focus of immunotherapy research, with inhibitors targeting this axis approved for the treatment of several solid tumors, including hepatocellular carcinoma, colorectal cancer, and GC.

PD-1, a member of the CD28 superfamily, is a type I transmembrane protein composed of 288 amino acids and is predominantly expressed on T cells (3). Its ligand, PD-L1, can be expressed on the surface of various tumor cell types (4). Upon engagement of PD - 1 by PD-L1, the intracellular domain of PD - 1 is phosphorylated at the tyrosine residue Y248 by Src family kinases, leading to the recruitment of SHP2 (Src homology region 2-containing phosphatase 2) (5). SHP2 subsequently inhibits T cell receptor (TCR) and CD3 co-stimulatory signaling pathways and downstream MAPK and PI3K/AKT cascades (6), the latter of which is critically involved in T cell survival and apoptosis regulation (7). Additionally, the recruited SHP2 enhances the lipid phosphatase activity of PTEN, which intercepts PI3K downstream signaling, further suppressing the PI3K/AKT pathway and downregulating Bcl-xl expression, thus promoting T cell apoptosis. Moreover, SHP2-mediated inhibition of tyrosine kinase phosphorylation downstream of TCR signaling, particularly phosphorylation of p38 and LAT, leads to further blockade of the MAPK pathway. These events collectively suppress the expression of genes associated with T cell proliferation and activity (8). In summary, binding of PD-L1 on tumor cells to PD - 1 on T cells induces T cell apoptosis, anergy, and exhaustion, thereby suppressing the activation, proliferation, and antitumor function of tumor antigen-specific CD8^+^ T cells, ultimately facilitating tumor immune escape. PD - 1/PD-L1 inhibitors work by disrupting this interaction, thereby restoring TCR and CD3-mediated co-stimulatory signaling, reactivating downstream PI3K/AKT and MAPK pathways, promoting T cell proliferation and activation, and ultimately counteracting tumor cell-mediated immunosuppression and inhibiting tumor growth and metastasis.

Recent studies have extended these insights by demonstrating that PD - 1 signaling also modulates T cell metabolism and epigenetic programming. Specifically, PD - 1 inhibits glycolysis and mitochondrial function through suppression of the PI3K/AKT/mTOR axis, leading to metabolic exhaustion of effector T cells (9). Moreover, PD - 1 signaling recruits DNA methyltransferase 1 (DNMT1) to silence effector gene loci, contributing to the establishment of epigenetic exhaustion programs in T cells, characterized by repressive chromatin and stable transcriptional inactivation of key cytokines and effector molecules (10). These effects synergistically drive T cell dysfunction, anergy, and exhaustion in the tumor microenvironment (TME). PD - 1/PD-L1 inhibitors work by disrupting this suppressive axis, thereby restoring TCR/CD3-mediated co-stimulatory signaling, reactivating downstream MAPK and AKT cascades, rescuing metabolic competence, and reversing exhaustion-related epigenetic modifications. This results in the restoration of cytotoxic T lymphocyte (CTL) activity, improved tumor infiltration, and enhanced antitumor immunity.

In the context of GC immunotherapy, several studies have confirmed the efficacy and safety of PD - 1/PD-L1 inhibitors. For instance, the ATTRACTION - 4 trial demonstrated that the addition of nivolumab to chemotherapy significantly improved progression-free survival (PFS) compared to chemotherapy alone in Asian patients with untreated, HER2-negative, unresectable advanced or recurrent gastric and gastroesophageal junction adenocarcinoma (hazard ratio [HR] 0.68 (11). Similarly, in another phase III clinical trial, patients with advanced GC who failed at least two prior chemotherapy regimens showed a median overall survival (OS) extension of 1.12 months in the nivolumab treatment group, with 1- and 3-year survival rates superior to those of the control group (12). These findings highlight the promising potential of nivolumab in improving survival outcomes with a favorable safety profile in GC patients. More recent data presented at major oncology congresses further reinforce the role of PD - 1 inhibitors in gastric cancer treatment. For example, final overall survival (OS) data from the CheckMate-649 trial, presented at ASCO 2023, demonstrated that nivolumab plus chemotherapy significantly prolonged OS compared to chemotherapy alone in patients with PD-L1 CPS ≥5 advanced gastric, gastroesophageal junction (GEJ), and esophageal adenocarcinoma. Similarly, updated findings from the KEYNOTE - 811 trial reported at ASCO 2024 confirmed that pembrolizumab combined with trastuzumab and chemotherapy significantly improved objective response rate (ORR) and progression-free survival (PFS) in HER2-positive gastric cancer. In addition, early-phase clinical trials presented at ASCO-GI 2024 indicated that the combination of PD - 1 inhibitors with FOLFOX as first-line treatment is feasible and may further expand the immunotherapy landscape for advanced GC (13).

### 2.2 Cytotoxic T lymphocyte-associated antigen-4

CTLA-4 is a negative regulatory molecule expressed on the membrane of T cells. It binds to B7 molecules on antigen-presenting cells (APCs), thereby suppressing T cell activation (14). Recent studies have focused extensively on bispecific antibodies targeting both PD - 1 and CTLA - 4, such as cadonilimab (codibody). For instance, the COMPASSION - 15 phase III clinical trial demonstrated that chemotherapy in combination with cadonilimab significantly improved PFS and OS compared to chemotherapy alone in treatment-naïve patients with HER2-negative locally advanced or metastatic gastric or gastroesophageal junction adenocarcinoma (HR = 0.62) (15).

### 2.3 Lymphocyte activation gene-3

LAG-3 is a promising immune checkpoint molecule, following PD - 1 and CTLA - 4. LAG - 3 promotes T cell exhaustion and inhibits T cell proliferation. Many tumor cells express fibrinogen-like protein 1 (FGL1), a major ligand of LAG - 3. The interaction between LAG - 3 and FGL1 leads to T cell exhaustion and impaired proliferation, thereby facilitating tumor progression (16). More than 20 LAG - 3-targeted inhibitors are currently under clinical investigation. Among them, relatlimab was approved by the U.S. Food and Drug Administration (FDA) in 2022 for use in combination with the PD - 1 inhibitor nivolumab at a fixed dose regimen for the treatment of unresectable or metastatic melanoma, marking LAG - 3 as the third immune checkpoint approved for clinical use (17, 18). In the context of GC, clinical trials such as NCT03044613 are evaluating the efficacy of relatlimab in combination with other ICIs, though data remain forthcoming. Clinical studies targeting LAG - 3 in GC remain limited, and its therapeutic potential warrants further investigation.

### 2.4 T cell immunoglobulin and mucin-domain containing-3

TIM-3 is predominantly expressed on antigen-specific CD8^+^ T cells, CD4^+^ T cells, and natural killer (NK) cells. Upon ligand binding, TIM - 3 inhibits the activation and maturation of immune effector cells and adaptive immune responses, thereby contributing to tumor cell proliferation and survival (19). A study analyzing the expression of inhibitory ligands in 365 patients with GC revealed that co-expression of ligands for PD - 1, TIM - 3, and LAG - 3 was the most frequent pattern (34.7%). This co-expression profile suggests that combinatorial immunotherapy targeting PD - 1, TIM - 3, and LAG - 3 may hold therapeutic promise for GC patients (20).

### 2.5 T cell immunoglobulin and ITIM domain

TIGIT exerts multiple immunosuppressive effects, including inhibition of NK cell effector function, suppression of dendritic cell (DC) maturation, promotion of macrophage polarization toward the M2 phenotype, and regulation of T cell differentiation. Preclinical evidence suggests that dual blockade of PD - 1 and TIGIT enhances the expansion of tumor antigen-specific CD8^+^ T cells, thereby eliciting antitumor activity (21). This concept has been supported by clinical findings in esophageal cancer, where dual inhibition of TIGIT and PD - 1 achieved improved objective response rate (ORR, 27.8%) and disease control rate (DCR, 50%) in immunotherapy-naïve patients (33.3% of whom had adenocarcinoma) (22). In GC, several ongoing studies are evaluating the efficacy and safety of combined anti-PD-1 and anti-TIGIT therapies. These include a phase II clinical trial (NCT04933227) assessing the combination of atezolizumab, tiragolumab, and XELOX as first-line treatment for patients with HER2-negative, unresectable, recurrent, or metastatic gastric or gastroesophageal junction (GEJ) adenocarcinoma. Another phase II trial (NCT05702229) is investigating AZD2936, a bispecific antibody targeting both PD - 1 and TIGIT, in combination with FOLFOX or XELOX as first-line therapy for HER2-negative unresectable or metastatic gastric or GEJ adenocarcinoma. The outcomes of these studies are awaited with interest.

Overall, current clinical evidence suggests that combination strategies involving ICIs may offer superior efficacy compared to monotherapies. Nonetheless, more robust evidence from large-scale, multicenter trials is needed to confirm these findings (**Table 1**).

**Table 1:** Recent advances in various immune checkpoint inhibitors.

Target	Recent research progress
PD-1/PD-L1	The U.S. Food and Drug Administration (FDA) has approved nivolumab and pembrolizumab, in combination with chemotherapy, for the treatment of advanced gastric cancer
CTLA-4	In gastric cancer, current studies predominantly focus on bispecific antibodies targeting both PD - 1 and CTLA - 4 (e.g., cadonilimab), which have demonstrated promising efficacy
LAG-3	Relatlimab received FDA approval in 2022 for use in fixed-dose combination with the PD - 1 inhibitor nivolumab for the treatment of unresectable or metastatic melanoma. In gastric cancer, relatlimab is under clinical investigation primarily in combination with other ICIs; however, trial results are not yet available
TIM-3	Combination immunotherapy targeting PD - 1, TIM - 3, and LAG - 3 has shown therapeutic potential in gastric cancer patients, particularly in cases with co-expression of multiple immune checkpoints
TIGIT	In esophageal cancer, dual blockade of TIGIT and PD - 1 has yielded superior clinical outcomes. Several ongoing trials are assessing the efficacy and safety of this approach in gastric cancer, and results are eagerly awaited

### 2.6 Resistance mechanisms to immune checkpoint inhibitors

Although ICIs have achieved notable clinical benefits in a subset of GC patients, both primary and acquired resistance remain major obstacles limiting their efficacy. Mechanistically, several tumor-intrinsic and extrinsic factors contribute to immune evasion and therapeutic failure.

Primary resistance often arises from impaired antigen presentation and insensitivity to immune effector signals. For example, mutations or epigenetic silencing of β2-microglobulin (B2M) lead to loss of MHC class I expression, thereby reducing tumor antigen visibility to CD8^+^ T cells. Additionally, inactivating alterations in the IFN-γ signaling pathway—particularly involving JAK1, JAK2, or STAT1—can diminish the transcriptional response required for T cell–mediated cytotoxicity (23).

Acquired resistance frequently emerges after initial treatment responses. One mechanism involves upregulation of alternative immune checkpoints, such as LAG - 3, TIM - 3, and TIGIT, which sustain T cell exhaustion despite PD - 1 blockade. Moreover, tumor cells may undergo immunoediting and clonal evolution, resulting in the loss of immunogenic neoantigens. Epigenetic remodeling and metabolic dysfunction within T cells, including DNMT1-mediated gene silencing and mTOR suppression, also contribute to sustained immune dysfunction and reduced therapeutic sensitivity (24).

Understanding these mechanisms is critical for the rational design of combination regimens and development of next-generation immunotherapies aimed at overcoming resistance.

## 3 Adoptive cell therapy

**Figure 2:**
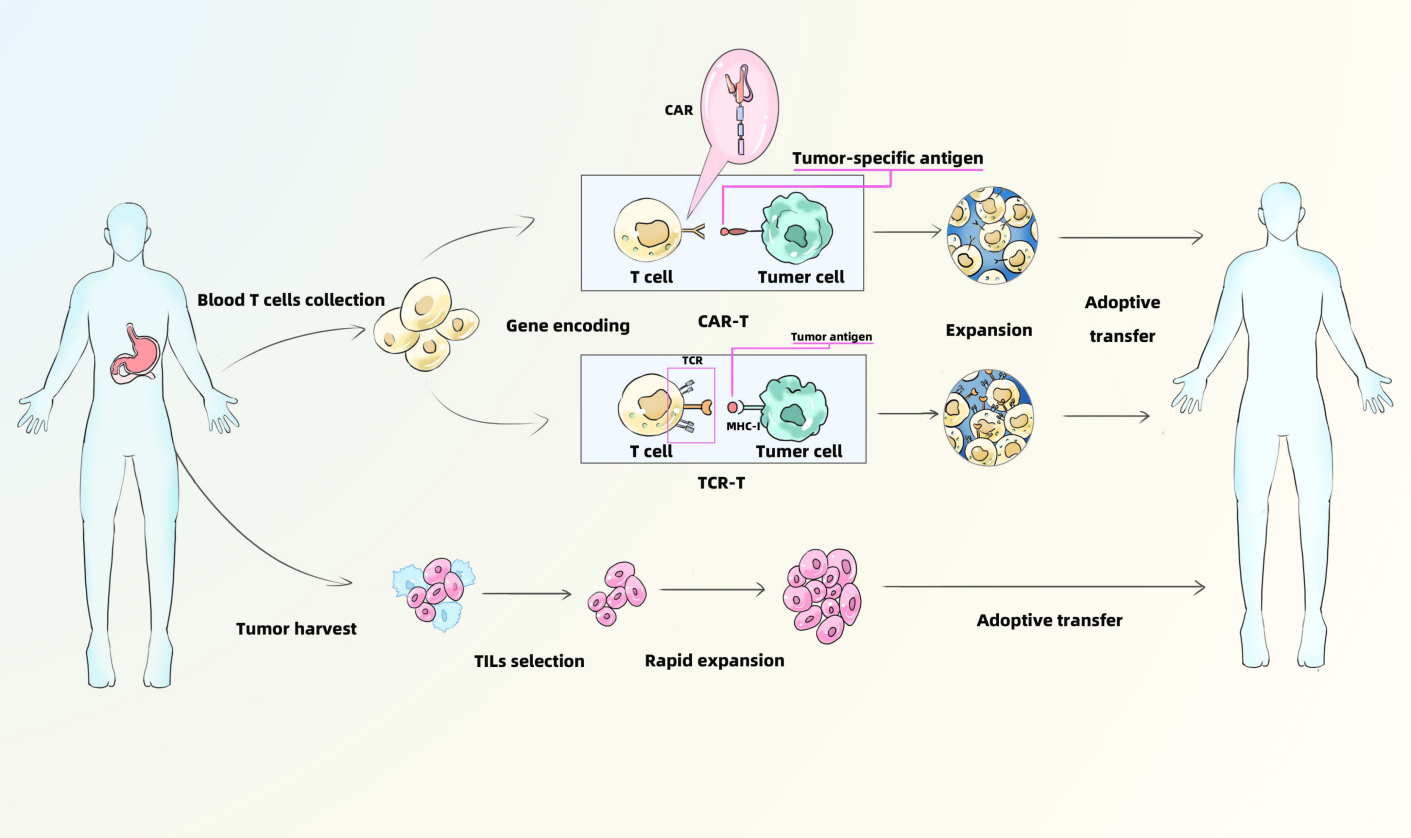
Mechanism of CAR-T、TCR-T and TILs. CAR-T, Chimeric Antigen Receptor T-Cell Immunotherapy; TCR-T, T Cell Receptor-Gene Engineered T Cells; TILs, Tumor Infiltrating Lymphocytes.

ACT is an emerging form of personalized immunotherapy that differs fundamentally from immune checkpoint inhibition. ACT involves isolating immune cells from the patient, followed by ex vivo culture, genetic modification or selection, and large-scale expansion. These modified immune cells can be engineered to express novel receptors—such as chimeric antigen receptors (CARs) (25)—which endow them with antitumor activity. Upon reinfusion into the patient, these cells are capable of specifically targeting and destroying tumor cells (**Figure 2**).

ACT has demonstrated significant therapeutic efficacy in various cancers, particularly melanoma and hematologic malignancies. Tumor-infiltrating lymphocyte (TIL) therapy has shown the potential to induce durable complete remission (CR) in patients with metastatic melanoma (26). Similarly, CD19-targeted CAR-T cell therapy has achieved impressive outcomes in relapsed/refractory acute lymphoblastic leukemia (ALL), with complete response (CR) rates reaching up to 90% (27). In refractory diffuse large B-cell lymphoma (DLBCL), CD19 CAR-T cell therapy has also shown promising efficacy, with an ORR of approximately 82% (28). However, in GC, the clinical application of ACT remains in its early stages. Several challenges hinder its efficacy in GC, including the high degree of tumor heterogeneity (29), an immunosuppressive TIME (30), and limited T cell infiltration (31). Despite these obstacles, ongoing research over recent years has led to notable progress in the development of ACT for GC. Multiple ACT modalities are being explored, including CAR-T cells, CAR-NK cells, T cell receptor-engineered T cells (TCR-T), and TILs. This section will discuss recent advancements in the application of various ACT strategies for the treatment of GC.

### 3.1 Chimeric antigen receptor T-cell immunotherapy

CAR-T cell therapy is currently one of the most extensively studied and promising approaches in cancer immunotherapy. It has been widely implemented in clinical trials and treatments for several malignancies. In GC, CAR-T therapy is undergoing rapid development, with active investigations targeting key tumor-associated antigens (TAAs). The following subsection will focus on several major CAR-T targets and research progress relevant to GC.

#### 3.1.1 Human epidermal growth factor receptor 2

Previous studies have demonstrated that HER2 is overexpressed in a substantial proportion of GC cases (32). HER2-targeted therapy has traditionally been implemented in the context of targeted agents. The pivotal ToGA trial confirmed that the addition of trastuzumab to chemotherapy provides significant clinical benefit for patients with HER2-positive advanced GC (33). With the growing prominence of immunotherapy, increasing attention has turned to HER2-targeted CAR-T cell therapy. One study reported that HER2-specific CAR-T cells effectively eliminated HER2-positive GC cells derived from patients and demonstrated markedly enhanced antitumor activity (34). Another preclinical study corroborated these findings in animal models (35). Collectively, these results suggest that HER2-directed CAR-T cell therapy holds considerable promise for the treatment of GC and warrants further investigation in future clinical studies.

#### 3.1.2 CLDN18.2

Claudin 18.2 (CLDN18.2) is highly expressed in GC and has been implicated in tumor initiation, progression, and metastasis. Its expression is also closely associated with patient prognosis, positioning CLDN18.2 as a critical emerging therapeutic target following HER2 (36). Preclinical studies have shown that CAR-T cells targeting CLDN18.2 can effectively suppress tumor growth without causing significant damage to normal tissues, including gastric mucosa (37). These findings suggest that CLDN18.2-specific CAR-T cells could represent a viable and selective treatment strategy for GC and other CLDN18.2-positive malignancies. Clinical evidence further supports this potential. In one case study, a patient with metastatic GC who had failed multiple prior treatments experienced a rapid expansion of CAR-T cells and a marked reduction in circulating tumor DNA (ctDNA) following two infusions of autologous CLDN18.2-targeted CAR-T cells (CT041), without developing severe adverse events (38). Similarly, results from a phase I clinical trial involving 37 patients with advanced gastric or gastroesophageal junction (G/GEJ) adenocarcinoma demonstrated promising efficacy: the ORR and DCR were 57.1% and 75.0%, respectively, with a 6-month OS rate of 81.2%. Notably, patients with higher CLDN18.2 expression appeared to derive greater clinical benefit (39). Although these findings are preliminary, they underscore the potential of CLDN18.2-directed CAR-T cell therapy as a novel and effective approach for GC, deserving further validation in larger clinical trials.

#### 3.1.3 Other targets

##### 3.1.3.1 Cellular mesenchymal epithelial transition factor

c-Met is frequently overexpressed in various malignancies and is known to promote the progression and metastasis of GC, contributing to poor clinical outcomes (40). As such, c-Met has emerged as a potential therapeutic target in GC. Recent studies have demonstrated that bifunctional CAR-T cells targeting both c-Met and PD - 1 exhibit enhanced antitumor activity compared to CAR-T cells targeting c-Met alone (41). This dual-targeting strategy represents a novel approach to augment CAR-T cell efficacy and overcome immune resistance mechanisms.

##### 3.1.3.2 Folate receptor 1

FOLR1 is overexpressed in GC tissues while exhibiting minimal expression in normal tissues, making it an attractive target for CAR-T cell therapy. One study reported that FOLR1-specific CAR-T cells effectively recognized and lysed FOLR1-positive GC cells, inducing cytotoxicity and subsequent tumor cell death (42).

##### 3.1.3.3 Epithelial cell adhesion molecule (EpCAM) and intercellular adhesion molecule-1

EpCAM is highly expressed in GC, and its inhibition has been shown to significantly suppress tumorigenesis and tumor progression (43). Similarly, ICAM - 1 is upregulated in GC and is associated with unfavorable prognosis (44). Notably, a recent study revealed that activation of EpCAM-specific CAR-T cells can lead to compensatory upregulation of ICAM - 1 on tumor cells, thereby increasing their susceptibility to dual-targeted CAR-T cells against both EpCAM and ICAM - 1. While monovalent EpCAM-targeted CAR-T therapy has demonstrated efficacy in eradicating various solid tumors, it has also been associated with tumor recurrence. In contrast, bispecific CAR-T cells targeting both EpCAM and ICAM - 1 showed enhanced antitumor activity, with the ability to prevent resistance and reduce relapse driven by heterogeneous or EpCAM-negative tumor subpopulations (45). These findings support the potential of multi-antigen-targeted CAR-T therapies to improve durability and potency in GC treatment.

In addition to identifying optimal tumor-associated antigens, recent research has also focused on enhancing the persistence and antitumor efficacy of CAR-T cells through epigenetic and metabolic modulation (46). For instance, targeted deletion or inhibition of the DNA demethylase TET2 has been shown to promote the formation of memory-like CAR-T cells, reduce exhaustion, and improve long-term persistence. Histone deacetylase inhibitors (HDACis) can similarly reprogram CAR-T cells toward a less exhausted, more cytotoxic phenotype. Moreover, spatial metabolic heterogeneity within the gastric tumor microenvironment—including regional hypoxia, glucose deprivation, and lactate accumulation—can severely impair CAR-T cell infiltration, survival, and effector function (47). Tumor regions with high glycolytic activity and acidic pH are known to promote immune exclusion and T cell exhaustion. To address these challenges, metabolic reprogramming strategies, such as enhancing oxidative phosphorylation or conferring resistance to lactic acid–induced dysfunction, are currently under investigation. Together, these emerging approaches may help overcome intrinsic limitations of CAR-T therapy in solid tumors like gastric cancer.

### 3.2 NK cell therapy

NK cells are key components of the innate immune system and play a crucial role in suppressing the initiation, progression, and metastasis of GC. NK cells exert their antitumor effects primarily through mechanisms such as antibody-dependent cellular cytotoxicity, the release of cytolytic granules, and the secretion of cytokines including interferon-gamma (IFN-γ) and tumor necrosis factor-alpha (TNF-α) (48, 49). In a preclinical study, a second-generation HER2-specific CARs (5.137.z) was introduced into NK - 92 cells to generate HER2-targeted CAR-NK cells, termed NK - 92/5.137.z cells. These engineered CAR-NK cells demonstrated enhanced cytotoxicity and higher cytokine secretion levels, resulting in significant antitumor efficacy in murine models bearing small HER2-positive tumor xenografts. However, in models with larger, multifocal tumors—more representative of clinical tumor burden—the monotherapy with NK - 92/5.137.z cells showed limited efficacy. Notably, the combination of NK - 92/5.137.z cells with the anti-angiogenic agent apatinib markedly improved therapeutic outcomes. This may be attributed to the elevated levels of angiogenesis typically observed in large tumors, which can form a physical and immunosuppressive barrier that impedes NK cell infiltration. Since sufficient intratumoral NK cell infiltration is critical for effective therapy, anti-angiogenic agents may enhance the efficacy of NK cell-based immunotherapy in larger tumor settings (50). In addition to genetically engineered NK cells, expanded and activated autologous NK cells have also been explored as a therapeutic strategy. A study utilizing autologous NK cell infusions in patients with advanced gastrointestinal malignancies reported favorable therapeutic outcomes with a good safety profile (51). Another study investigated the combination of expanded and activated autologous NK cells with trastuzumab in HER2-positive GC. The results revealed that NK cells exhibited strong cytotoxic activity against tumor cells targeted by trastuzumab (52). In summary, while NK cell-based therapies for GC remain in the early stages of development, emerging preclinical and clinical findings suggest promising potential. Further research is warranted to comprehensively evaluate their efficacy and safety in larger, controlled trials.

### 3.3 TCR-T

Compared to CAR-T therapy, TCR-T possess the ability to proliferate more effectively under high antigen pressure and are not limited to antigens presented on the cell surface. Instead, they can flexibly recognize a broad spectrum of TAAs presented by major histocompatibility complex (MHC) molecules, while exhibiting minimal off-tumor cytotoxicity toward normal tissues (53, 54). Clinical trials in hematologic malignancies and select solid tumors have demonstrated the ability of TCR-T cells to mediate tumor lysis and eradication with promising therapeutic efficacy (55). In GC, the efficacy of TCR-T therapy remains to be fully elucidated. Despite its considerable immunotherapeutic potential, TCR-T therapy is associated with certain limitations. For instance, TCRs with high affinity may induce off-target toxicities, whereas low-affinity TCRs often fail to achieve sufficient therapeutic efficacy (56). Therefore, thorough pre-treatment evaluation and continuous monitoring during therapy are essential to ensure treatment safety and effectiveness.

### 3.4 TILs

TIL therapy involves isolating T cells from resected tumor tissue, followed by ex vivo selection and expansion before reinfusion into the patient to amplify the antitumor immune response. This approach represents a highly personalized form of cancer immunotherapy and has shown notable efficacy in melanoma (57) and cervical cancer (58). In GC, high TIL infiltration has been associated with improved prognosis, and evidence suggests that reinfusion of expanded autologous TILs may enhance therapeutic outcomes (59). However, no reliable clinical trial data are currently available for TIL-based therapy in GC, and its clinical efficacy in this context requires further investigation (**Table 2**).

**Table 2:** Advantages and disadvantages of various ACT modalities.

ACT modality	Advantages	Limitations
CAR-T	Highly specific and personalized therapy	Potential for severe immune-related adverse events
NK Cell Therapy	Applicable across a wide range of tumor types; lower risk of immune-related toxicity	Relatively lower specificity and therapeutic efficacy; requires frequent infusion of large numbers of NK cells to maintain effect
TCR-T	Capable of recognizing a broad spectrum of tumor antigens; suitable for tumors unresponsive to CAR-T therapy	High specificity for antigen-HLA complexes is required; risk of off-target toxicity
TILs	Applicable to various tumor types	Inconsistent clinical efficacy; labor-intensive and time-consuming cell isolation and expansion process

## 4 Tumor vaccines

Tumor vaccines represent an emerging strategy in the immunotherapy landscape of GC, aiming to activate the host immune system against TAAs to elicit a robust antitumor immune response. Vaccine platforms under investigation include DC vaccines, therapeutic B-cell epitope vaccines, mRNA vaccines, and OV-based vaccines.

### 4.1 DC vaccines

Recent case reports have highlighted the clinical potential of DC-based vaccines in advanced GC. In one notable case, a patient with metastatic GC experienced complete tumor regression sustained for 25 months following the first administration of a personalized neoantigen-loaded monocyte-derived DC vaccine (Neo-MoDC) in combination with PD - 1 blockade (60). This represents the first reported instance of durable and complete tumor regression in GC using a neoantigen-based DC vaccine in conjunction with PD - 1 inhibition—a milestone in the field. Further large-scale studies are warranted to validate the efficacy and safety of Neo-MoDC vaccines in a broader patient population.

### 4.2 Therapeutic B cell epitope vaccines

Therapeutic vaccines targeting B-cell epitopes have also demonstrated encouraging results in GC, particularly in HER2-overexpressing tumors. A phase Ib clinical trial evaluating HER-Vaxx (IMU - 131), a B-cell epitope vaccine targeting HER2, in patients with advanced HER2-positive GC reported promising outcomes (61). Among the 14 participants, tumor reduction and vaccine-specific immune responses were observed in 11 patients, including one CR, five partial responses (PR), and four cases of stable disease (SD). No vaccine-related serious adverse events were reported, indicating a favorable safety profile. More recently, results from a phase II clinical trial further confirmed the clinical potential of HER-Vaxx (62). Compared to chemotherapy alone, the combination of HER-Vaxx with chemotherapy led to a 40% improvement in OS (HR: 0.60; median OS: 13.9 months vs. 8.31 months). Tumor size decreased by an average of 30% from baseline in the combination group, compared to a 10% reduction in the chemotherapy-only group. These findings suggest that HER2-specific antibody responses induced by HER-Vaxx are significantly associated with tumor shrinkage and improved immune function. Furthermore, the favorable safety and efficacy outcomes observed in both phase I and II trials support continued development of this therapeutic vaccine strategy. However, several translational and regulatory challenges must be addressed before HER-Vaxx can be widely adopted in clinical practice. One issue is inter-individual variability in vaccine-induced antibody titers, which may affect consistency of treatment responses and necessitate personalized immunomonitoring or booster adjustments. In addition, as a peptide-based formulation, HER-Vaxx requires cold-chain storage and transport, which may limit accessibility in resource-limited settings. From a regulatory standpoint, standardization of peptide vaccine manufacturing, batch-to-batch consistency, and quality control remain critical hurdles for large-scale commercialization (63).

### 4.3 mRNA vaccines

The mechanism of action of mRNA vaccines involves identifying optimal TAAs through genomic sequencing of tumor tissue, followed by *in vitro* synthesis of the corresponding mRNA sequences. These mRNA molecules are then delivered into the body, where they are translated into target antigens, thereby enhancing antigen presentation and stimulating antitumor immunity. A recent study in pancreatic ductal adenocarcinoma demonstrated that patients receiving an mRNA-based personalized vaccine exhibited increased numbers of tumor-specific T cells and prolonged median recurrence-free survival compared to those who did not receive the vaccine (64). Despite their promise, mRNA vaccines face unique challenges in gastric cancer. These include inefficient delivery to antigen-presenting cells (APCs), immune tolerance within the gastric microenvironment, and limited accuracy in neoantigen prediction due to the typically low tumor mutational burden in GC. Moreover, the dense stroma and immunosuppressive cellular composition of GC may further limit vaccine efficacy. Although no clinical data have been published for mRNA vaccines in GC, preclinical studies using lipid nanoparticle (LNP)-formulated mRNA vaccines targeting GC antigens have demonstrated induction of antigen-specific T cell responses and delayed tumor progression in murine models.

In China, clinical trials have been initiated for two mRNA-based cancer vaccines—XH101, a neoantigen-based mRNA vaccine injection, and EVM16, a personalized tumor vaccine—but their results in GC remain unpublished. These efforts highlight the need for continued development of delivery systems and combination strategies (e.g., checkpoint inhibitors or TLR agonists) to overcome GC-specific immunological barriers.

### 4.4 Oncolytic virus vaccines

OV vaccines exert antitumor effects by selectively infecting and lysing tumor cells, while simultaneously activating systemic antitumor immune responses. In patients with residual or recurrent glioblastoma, the genetically modified herpes simplex virus type 1 (HSV - 1)-based OV G47Δ has shown significant therapeutic efficacy and was approved as the first OV product in Japan (65). In the context of solid tumors, a study by Zhang et al. (66) demonstrated that intratumoral injection of OH_2_—a genetically engineered oncolytic HSV - 2—induced durable antitumor responses in patients with metastatic esophageal and rectal cancers. However, no data are yet available for its application in GC. Additional clinical studies are required to evaluate the therapeutic efficacy and safety of OV vaccines in this setting.

However, the application of OV vaccines in gastric cancer presents specific challenges. These include inefficient viral penetration due to mucus barriers, low expression of viral entry receptors on GC cells, and a highly immunosuppressive tumor microenvironment that impedes effective viral replication and immune priming. Preclinical studies using GC-bearing mouse models have shown that intratumorally injection of engineered herpesvirus or adenovirus can induce local tumor regression and enhance CD8^+^ T cell infiltration, particularly when combined with immune checkpoint blockade or radiation therapy. Although clinical data for OV vaccines in GC are currently lacking, these experimental results provide a compelling rationale for their further development and clinical evaluation in gastric cancer immunotherapy.

Despite the promising results described above, tumor vaccine development in gastric cancer still faces significant translational hurdles. One major barrier is the limited accuracy of neoantigen prediction algorithms, especially in GC, which typically exhibits low tumor mutational burden. This can result in poor immunogenicity or immune escape due to antigen loss (67). In addition, various delivery platforms—including peptide-, DC-, mRNA-, and virus-based systems—are constrained by instability, low efficiency of antigen uptake, or manufacturing complexity (68). Tumor heterogeneity and the immunosuppressive tumor microenvironment also contribute to immune tolerance, limiting the magnitude and durability of vaccine-induced responses. Moreover, as tumors evolve under immune pressure, immune escape variants may emerge that are no longer targeted by the original vaccine. To overcome these obstacles, integrative strategies are under development. These include AI-assisted neoantigen prioritization, nanoparticle-based delivery systems to enhance APC targeting, and combination approaches with checkpoint inhibitors or adjuvants to boost vaccine efficacy. Furthermore, advances in spatial transcriptomics and single-cell sequencing can help map tumor heterogeneity and optimize vaccine design (69). These efforts are essential to unlock the full therapeutic potential of tumor vaccines in GC.

In summary, these emerging studies highlight the immense potential of tumor vaccines as a novel therapeutic strategy. Continued investigation is warranted to further explore their application in GC immunotherapy (**Table 3**).

**Table 3:** Recent advances in tumor vaccine research.

Vaccine type	Recent research progress
Dendritic Cell Vaccine	In a reported case of advanced metastatic gastric cancer, complete tumor regression lasting up to 25 months was achieved following initial treatment with a personalized neoantigen-loaded monocyte-derived dendritic cell vaccine (Neo-MoDC) combined with PD - 1 blockade. This represents the first successful case of such therapy in gastric cancer.
Therapeutic B Cell Epitope Vaccine	A phase II clinical trial of HER-Vaxx (IMU - 131), a therapeutic B cell epitope vaccine for patients with advanced HER2-overexpressing gastric cancer, demonstrated that vaccine-induced HER2-specific antibody responses were significantly associated with tumor regression and improved immunologic function. The addition of chemotherapy further enhanced therapeutic efficacy.
mRNA Vaccine	While mRNA-based vaccines have shown promising therapeutic effects in pancreatic ductal adenocarcinoma, clinical trial data for their use in gastric cancer are currently lacking.
Oncolytic Virus Vaccine	Oncolytic virus Vaccine has demonstrated antitumor activity in patients with metastatic esophageal and rectal cancers; however, there are currently no clinical data available regarding its use in gastric cancer.

## 5 Immunotherapy-related biomarkers

**Figure 3:**
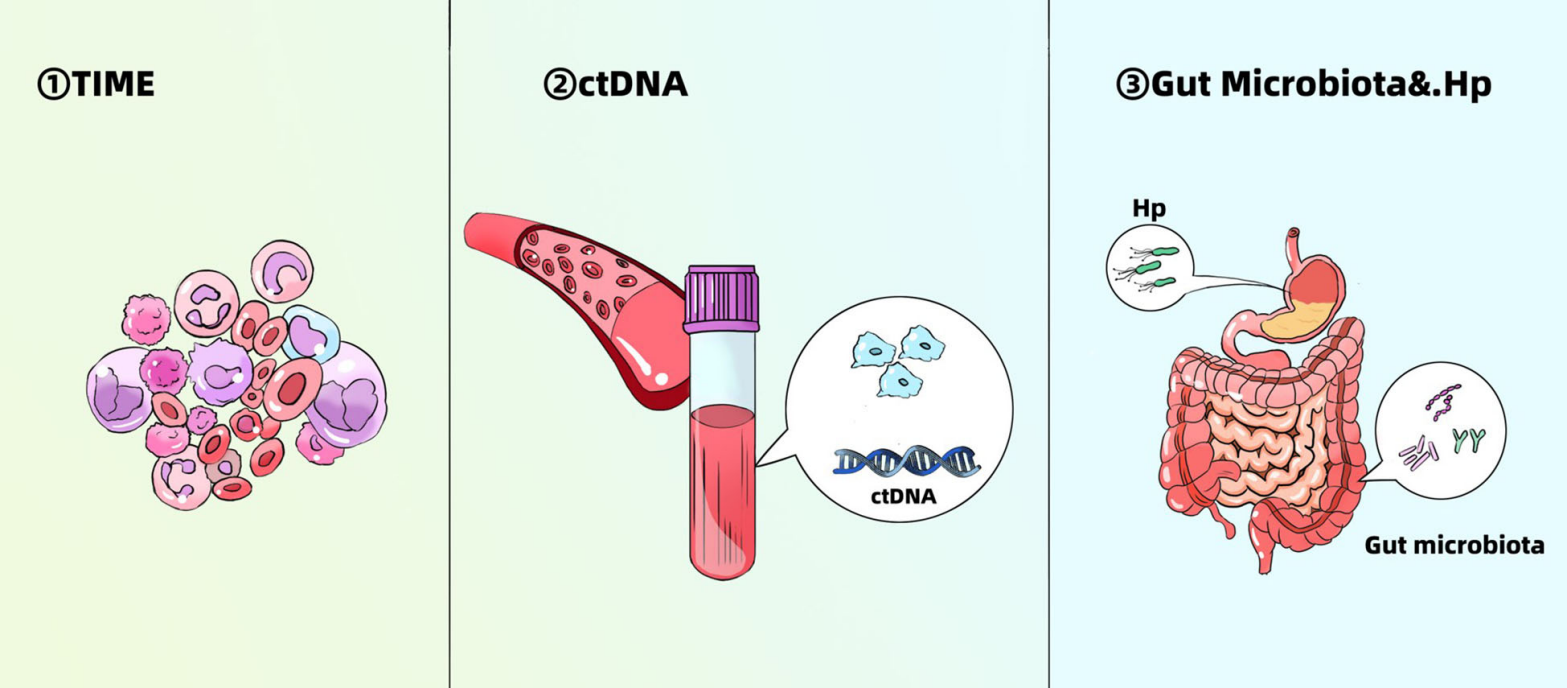
Emerging biomarkers for predicting efficacy of immunotherapy in gastric cancer. TIME, tumor immune microenvironment; Hp, Helicobacter pylori.

To predict and assess the efficacy of immunotherapy in GC patients, reliable biomarkers are essential. Currently, several biomarkers—such as microsatellite instability-high (MSI-H), programmed death-ligand 1 (PD-L1), and tumor mutation burden (TMB)—are relatively well-established for predicting response to immunotherapy in GC (70, 71). However, each of these biomarkers has inherent limitations, and their predictive value can be further refined through stratification. Therefore, this section focuses on summarizing several emerging and potentially more robust predictive biomarkers, aiming to provide additional guidance for clinical decision-making (**Figure 3**).

### 5.1 TIME

The TIME plays a critical role in immune evasion and the development of therapeutic resistance in cancer. In GC, high levels of TILs, particularly CD8^+^ T cells, are generally associated with favorable prognosis and improved response to immunotherapy (72). Conversely, increased infiltration of immunosuppressive cells such as regulatory T cells (Tregs) and myeloid-derived suppressor cells (MDSCs) often correlates with poor outcomes and limited therapeutic benefit (73). Notably, Zeng et al. (74) analyzed immune cell compositions within the TIME across 1,524 GC cases and proposed a novel TIME scoring system to quantify the immune status of tumor tissues. Their findings demonstrated that high TIME scores characterize immune-activated tumor subtypes and are significantly associated with better prognosis (HR = 0.42; 95% confidence interval [CI]: 0.33 - 0.54; *P* < 0.001), while low TIME scores are indicative of immunosuppressive tumor profiles and poor outcomes. Subsequently, the predictive value of the TIME score was validated in a multicenter clinical cohort, confirming its potential to forecast immunotherapy efficacy in GC (75). These findings suggest that the TIME represents a promising predictive biomarker for immunotherapy responsiveness in GC. Further exploration of TIME-related signatures may contribute to the development of more precise and individualized immunotherapeutic strategies. Recent advances in artificial intelligence (AI) have enabled the development of machine learning models that incorporate spatial transcriptomic and TIME features to predict immunotherapy response in gastric cancer. For example, Zeng et al. (2021) established a TIME-based signature using a random forest classifier that stratified patients by immune subtype and accurately predicted response to checkpoint inhibitors (75).

In addition, emerging high-resolution technologies such as single-cell RNA sequencing (scRNA-seq) and spatial transcriptomics have provided unprecedented insights into the intratumoral immune landscape of GC. scRNA-seq enables the identification of rare or functionally distinct immune subsets—such as exhausted CD8^+^ T cells and immunosuppressive macrophage clusters—that contribute to immune evasion and therapeutic resistance (76). Meanwhile, spatial transcriptomics allows for the preservation of spatial context and reveals localized immune exclusion patterns within the TIME, which are not captured by bulk profiling. These tools have been successfully used to map tumor-immune interactions and identify novel TIME phenotypes predictive of ICI response. Integrating scRNA-seq and spatial transcriptomic data with clinical and genomic features is expected to further refine patient stratification and guide precision immunotherapy in gastric cancer (77).

### 5.2 ctDNA

ctDNA has been reported to reflect the expression level of PD-L1 in tumor tissues indirectly, predict prognosis, identify resistance to targeted therapies, and monitor tumor recurrence (78). A study by Jin et al. (79), involving 46 patients with advanced GC receiving PD - 1 inhibitor plus chemotherapy, found a correlation between ctDNA detection and survival outcomes. Specifically, patients who achieved ctDNA clearance after treatment exhibited significantly prolonged median survival (*P* = 0.025), suggesting that a post-treatment decrease in ctDNA may serve as a predictive marker of immunotherapy efficacy. Another clinical study of 61 GC patients similarly showed that a reduction in ctDNA levels was associated with improved prognosis (80), thereby reinforcing the predictive value of dynamic ctDNA monitoring. These findings indicate that ctDNA is a promising biomarker for assessing immunotherapeutic response and tracking disease recurrence in GC. Moreover, Phase II-III clinical trials (e.g., NCT04053725) are currently investigating the utility of serial ctDNA measurements combined with AI-based analytics for early prediction of immunotherapy efficacy in GC. These studies demonstrate emerging clinical feasibility of AI-assisted time-resolved biomarkers to inform precision immunotherapy decisions.

### 5.3 Gut microbiota and *Helicobacter pylori*

The gut microbiota has emerged as a key regulator of antitumor immunity during immunotherapy, capable of modulating host immune responses and influencing therapeutic outcomes (81). A recent study identified several bacterial species within the gut microbiome that enhance antitumor immunity by downregulating the expression of PD-L2 and its binding partner, RGMb. PD-L2, like PD-L1, is a ligand for PD - 1, and its interaction with PD - 1 suppresses immune cell function; in addition, PD-L2 can bind to RGMb. This discovery suggests a novel immunotherapeutic strategy—manipulation of the gut microbiota to suppress the PD-L2–RGMb axis—which may benefit patients who are non-responsive to PD - 1 blockade (82). These findings highlight the potential of the gut microbiome as a predictive biomarker for immunotherapy, warranting further investigation.

Moreover, *H. pylori* infection may also serve as a predictor of response to immunotherapy in GC. In a study evaluating PD - 1 inhibitor therapy in GC, patients without *H. pylori* infection had significantly longer OS than those who were *H. pylori* positive (17.5 months vs. 6.2 months). Additionally, *H. pylori*-positive patients were more prone to adverse events (83). These findings suggest that *H. pylori* status could represent a clinically relevant biomarker for predicting both efficacy and safety of ICIs in GC (**Table 4**).

**Table 4:** Characteristics of predictive biomarkers for immunotherapy efficacy in gastric cancer.

Biomarker	Advantages	Limitations
TIME	Provides a comprehensive assessment of the immune characteristics within the tumor microenvironment	Time-consuming and relatively expensive to analyze
ctDNA	Enables real-time, dynamic monitoring of treatment response and minimal residual disease	High detection costs, less accurate in gastric cancer than in colon cancer
Gut Microbiota	Associated with regulation of PD-L2 and its binding partner RGMb	Limited clinical research available in the context of gastric cancer immunotherapy
*Helicobacter pylori*	Correlated with differential outcomes in gastric cancer patients receiving anti-PD-1 therapy	Requires validation in large-scale, multicenter prospective clinical trials

## 6 Conclusion and future perspectives

Research and clinical application of immunotherapy in GC are advancing rapidly, offering new hope for patients, particularly those with advanced or metastatic disease who are ineligible for surgical intervention.

Nonetheless, several challenges remain. Due to the high degree of molecular and immunological heterogeneity of GC, as well as the immunosuppressive nature of the gastric TIME, immunotherapeutic approaches are not universally effective. For instance, CAR-T cell therapy may fail to achieve clinical efficacy due to antigen heterogeneity and may even inadvertently target healthy tissues. This raises a critical question in the field: how can immunotherapy be tailored to deliver optimal efficacy for individual patients? Achieving precision in immunotherapy remains a central challenge. To address this, reliable tools are needed to predict and evaluate patient responses. As reviewed in Section 5, emerging

biomarkers such as TIME characteristics, ctDNA, and gut microbiota profiles show promise in predicting immunotherapy outcomes. These can be integrated with established biomarkers like MSI-H, PD-L1 expression, and TMB, providing a more comprehensive approach to treatment stratification in clinical settings. Looking ahead, efforts should be directed toward the identification of novel immunological targets and the rational combination of existing immunotherapeutic strategies. Our review of recent advances highlights that combination approaches—such as dual immune checkpoint blockade, bispecific CAR constructs, and the integration of immunotherapy with targeted agents—appear to offer superior efficacy and precision compared to monotherapies.

Future research should thus prioritize the integration of biomarker-informed patient stratification to design individualized combination immunotherapy regimens. This includes determining the optimal therapeutic agents, dosing strategies, and synergistic drug combinations to maximize antitumor efficacy while minimizing immune-related adverse events. In parallel, artificial intelligence (AI)-assisted strategies are increasingly being developed to support individualized immunotherapy decision-making. Recent advances in machine learning and computational modeling have enabled the integration of high-dimensional data—including TIME features, ctDNA dynamics, spatial transcriptomics, and clinical variables—to predict patient response to immune checkpoint inhibitors. AI-driven platforms can also assist in identifying optimal dosing schedules, forecasting immune-related adverse events, and selecting synergistic drug combinations (84, 85). As these tools evolve, their incorporation into clinical practice is expected to facilitate dynamic, data-informed optimization of immunotherapy regimens, thereby enhancing treatment precision and patient outcomes in gastric cancer. In addition to these technological advances, recent findings from ASCO-GI 2025 have provided important insights into novel immunotherapeutic strategies and clinical progress in gastric cancer. The phase III DRAGON - 01 trial demonstrated the efficacy of combining intraperitoneal and intravenous paclitaxel with S - 1 for patients with peritoneal metastasis, offering a promising direction for locoregional treatment (86). Meanwhile, subgroup analysis from the CABINET trial suggested that cabozantinib may serve as a viable option for gastrointestinal neuroendocrine tumors progressing after prior therapy (87). These latest results further expand the therapeutic options and provide new data that support precision-guided approaches in the immunotherapy era.

In addition to tumor-intrinsic factors, population-level differences have also emerged as important determinants of immunotherapy response in gastric cancer. Notably, the response to immunotherapy in gastric cancer exhibits significant geographic and ethnic variation (88). Clinical trials have consistently demonstrated higher objective response rates and improved survival outcomes among Asian patients compared to their non-Asian counterparts (89). This disparity may be attributed to multiple factors, including differences in tumor mutational burden (TMB), Epstein-Barr virus (EBV) positivity rates, microsatellite instability (MSI) prevalence, and HLA allele distribution (90). For example, MSI-H and EBV-positive gastric cancers—both associated with better immunotherapy responses—are more commonly observed in East Asian populations.

Besides, recent studies have increasingly highlighted the critical role of the gut microbiota in shaping systemic immune responses and influencing the efficacy of cancer immunotherapies (91, 92). Commensal bacteria modulate host immunity by regulating dendritic cell maturation, enhancing antigen presentation, and promoting the expansion of effector T cells. In gastric cancer, specific microbial signatures—such as enrichment of Helicobacter pylori, Fusobacterium, and Peptostreptococcus—have been associated with immune suppression and tumor progression. Conversely, beneficial microbes like *Akkermansia muciniphila* and *Faecalibacterium prausnitzii* have been linked to enhanced responses to immune checkpoint inhibitors (ICIs), partly through the modulation of gut-derived metabolites and gut-barrier integrity (93). Moreover, fecal microbiota transplantation (FMT) and microbial engineering are being investigated as potential adjuvant strategies to overcome resistance to immunotherapy. Integrating microbiome profiling into patient stratification could improve immunotherapeutic outcomes in gastric cancer and may offer new avenues for combination therapies. Despite the therapeutic promise of ICIs and combination strategies, immune-related adverse events (irAEs) remain a significant clinical challenge. These toxicities result from immune system hyperactivation and can affect multiple organs, including the skin, liver, lungs, and gastrointestinal tract. The underlying mechanisms include loss of peripheral tolerance, expansion of autoreactive T cells, and cytokine dysregulation. Recent studies have identified several predictive biomarkers for irAEs, such as elevated serum IL - 17 levels, specific HLA genotypes, and characteristic gut microbiota profiles—particularly decreased abundance of Faecalibacterium prausnitzii and increased Bacteroides fragilis abundance (94). To minimize the risk of irAEs while maintaining efficacy, strategies such as biomarker-informed patient stratification, optimized treatment sequencing, and pre-emptive immunomodulation are under investigation. Integration of AI-based predictive models combining clinical, molecular, and microbial features may further enhance early detection and management of irAEs (95). As the field advances, balancing antitumor efficacy and immune toxicity will be critical to realizing the full potential of personalized immunotherapy.

In conclusion, immunotherapy is redefining the therapeutic landscape of GC, with the potential to deliver more personalized and precise treatment modalities. Continued clinical research aimed at overcoming current limitations and optimizing therapeutic protocols will be essential to further improve patient survival and quality of life.
